# Organ-on-a-Chip Models of the Female Reproductive System: Current Progress and Future Perspectives

**DOI:** 10.3390/mi16101125

**Published:** 2025-09-30

**Authors:** Min Pan, Huike Chen, Kai Deng, Ke Xiao

**Affiliations:** 1 School of Medicine, Southeast University, Nanjing 210009, China; 2 State Key Laboratory of Digital Medical Engineering, Southeast University, Nanjing 211189, China; 3 Clinical Center of Reproductive Medicine, State Key Laboratory of Reproductive Medicine, First Affiliated Hospital, Nanjing Medical University, Nanjing 210029, China; 4 School of Biological Science and Medical Engineering, Southeast University, Nanjing 211189, China

**Keywords:** organ-on-a-chip, female reproductive system, construction methods, physiological simulation, multi-organ-chip

## Abstract

The female reproductive system represents a highly complex regulatory network governing critical physiological functions, encompassing reproductive capacity and endocrine regulation that maintains female physiological homeostasis. The in vitro simulation system provides a novel tool for biomedical research and can be used as physiological and pathological models to study the female reproductive system. Recent advances in this technology have evolved from 2D and 3D printing to organ-on-a-chip (OOC) and microfluidic systems, which has emerged as a transformative platform for modeling the female reproductive system. These microphysiological systems integrate microfluidics, 3D cell culture, and biomimetic scaffolds to replicate key functional aspects of reproductive organs and tissues. They have enabled precise simulation of hormonal regulation, embryo-endometrium interactions, and disease mechanisms such as endometriosis and gynecologic cancers. This review highlights the current state of female reproductive OOCs, including ovary-, uterus-, and fallopian tube-on-a-chip system, their applications in assisted reproduction and disease modeling, and the technological hurdles to their widespread application. Though significant barriers remain in scaling OOCs for high-throughput drug screening, standardizing protocols for clinical applications, and validating their predictive value against human patient outcomes, OOCs have emerged as a transformative platform to model complex pathologies, offering unprecedented insights into disease mechanisms and personalized therapeutic interventions. Future directions, including multi-organ integration for systemic reproductive modeling, incorporation of microbiome interactions, and clinical translation for mechanisms of drug action, will facilitate unprecedented insights into reproductive physiology and pathology.

## 1. Introduction

The female reproductive system orchestrates critical functions including gametogenesis, endocrine regulation, and pregnancy maintenance, which impacts both individual wellbeing and societal sustainability [1,2]. Women’s reproductive health is a core pillar of the public health system, which not only concerns the quality of individual life, but also directly affects the demographic structure and sustainable development. Globally, gynecological diseases cause over 3 million annual deaths, with emerging pandemiological patterns transcending geographical and demographic boundaries [3]. Gynecological tumors are one of the most prevalent malignant tumors in women worldwide, mainly including endometrial, cervical, and ovarian cancer [4]. Meanwhile, infertility has become an important public health problem that threatens reproductive health, and female reproductive disorders accounting for over 40% of infertility cases. Most research has relied on traditional 2D cell culture and animal models to build female reproductive organ models, which mimic certain features in the human body. However, 2D cell culture models cannot implement the complex 3D morphology and arrangement of cells in the female reproductive system [5,6], while animal models simulating the human body usually exhibit large variations and cannot accurately reflect the onset and progression of disease in the human body because of species difference [7,8,9]. For instance, rodent models of polycystic ovary syndrome (PCOS) fail to replicate human ovarian morphological features like stromal hyperplasia, and primate models lack evidence for primary ovarian defects identical to human [10]. For endometriosis, surgically induced lesions shrink in pigs and sheep but expand in dogs, diverging from human lesion progression patterns [11]. Therefore, these models have inherent limitations in replicating the complex in vivo environment, particularly the systemic endocrine regulation. All these issues restrict the study of female reproductive system, hence in vitro models are urgently needed to study female reproduction.

These limitations have spurred the advancement of novel modeling approaches, thereby bringing innovative technologies such as organoids and OOC to the forefront of research. As 3D tissue-like constructs generated through in vitro stem cell culture, organoids are capable of recapitulating the cellular composition and partial structural functionalities of multiple organs. 3D printing has become a core high-fidelity fabrication method for OOC, enabling advanced modeling of the architecturally complex and hormonally sensitive female reproductive system. Pioneering work by Juncker and Folch has pioneered PDMS-alternative hydrophilic photoresins and high-resolution stereolithography printing, respectively, overcoming key limitations in material adsorption and complex structure fabrication to enable high-fidelity female reproductive organ-on-chip models [12,13,14].

Though solving some core obstacles of material adsorption and complex structure fabrication, their capacity to simulate dynamic physiological microenvironments remains limited. Building upon the foundational framework of organoids, OOC technology has achieved further pivotal breakthroughs and emerged as a transformative platform in biomedical research. It not only accurately reproduces the 3D structural characteristics of target tissues/organs but also enables precise modulation of dynamic biological factors, including fluid flow and mechanical forces, through microfluidic-based engineering. This versatility allows OOC to address key limitations of traditional models, offering unprecedented insights into disease mechanisms and supporting the development of personalized therapeutic interventions.

In the specific realm of the female reproductive system, OOC technology has demonstrated remarkable applicability. For instance, it facilitates the establishment of multi-layered cellular architectures analogous to those observed in vivo [15,16,17,18], closely mimicking the structural complexity of female reproductive organs. Ovarian organoids can replicate the fundamental architecture of follicular development. Building on this, OOC technology has emerged as a transformative platform to simulate complex reproductive pathologies, offering unprecedented insights into disease mechanisms and personalized therapeutic interventions. The endometrial chip serves as a representative example: it not only recapitulates the morphological features and spatial organization of epithelial and stromal cell layers [19,20,21], but also simulates in vivo cyclical physiological processes via integrated perfusion systems. Specifically, simulation of fluid dynamics within fallopian tube chips supports investigations into ovum transport and fertilization mechanisms, while dynamic culture protocols for endometrial chips provide a physiologically relevant in vitro platform to dissect the molecular regulatory networks underlying the menstrual cycle [22,23]. Beyond basic research, endometrial-on-a-chip has advanced in vitro fertilization studies by optimizing embryo implantation timing, and tumor-on-chip models enable personalized drug screening for ovarian and cervical cancers. Furthermore, the advent of multi-organ-chip (MOC) technology represents a significant leap forward. By integrating microfluidics, bioengineering, and organoid technologies, MOCs build on the strengths of OOCs, enabling the construction of more complex and interconnected in vitro models that closely mimic the interactions between different reproductive organs. This technology allows for the simultaneous study of multiple organs and their coordinated physiological responses, offering new opportunities to explore the complex regulatory mechanisms in female reproductive processes and to assess the impact of drugs on multiple organs, thus providing a more comprehensive and realistic research platform for female reproductive health studies.

Notably, although OOC technology shows immense potential for advancing female reproductive research, and there have been existing reviews discussing the development of microengineered female reproductive organ models with relevance to drug delivery and discovery. However, it still lacks comprehensive review systematically summarizing construction methods, physiological and anatomical simulation, and application across diverse female reproductive organs remains. Moreover, by focusing resource that bridges engineering innovations with clinical problems in reproductive medicine, this review also provided updated advances in clinical applications like OOC optimizing embryo implantation timing in assisted reproduction, fallopian tube-on-a-chip solving polyspermic fertilization in in vitro fertilization (IVF), and ovarian tumor microenvironment chip (OTME-Chip)’s antimetastatic drug screening details. To address this gap, this review systematically summarizes the recent advancements in OOC models for the female reproductive system, with a focus on its accomplishments in constructing organ-specific chip models. By synthesizing current progress, this review provides novel technical insights for the reproductive biology and medicine, and offers physiologically relevant models to enhance drug development and toxicity assessment.

## 2. Organ-on-a-Chips in the Female Reproductive System

**Figure 1:**
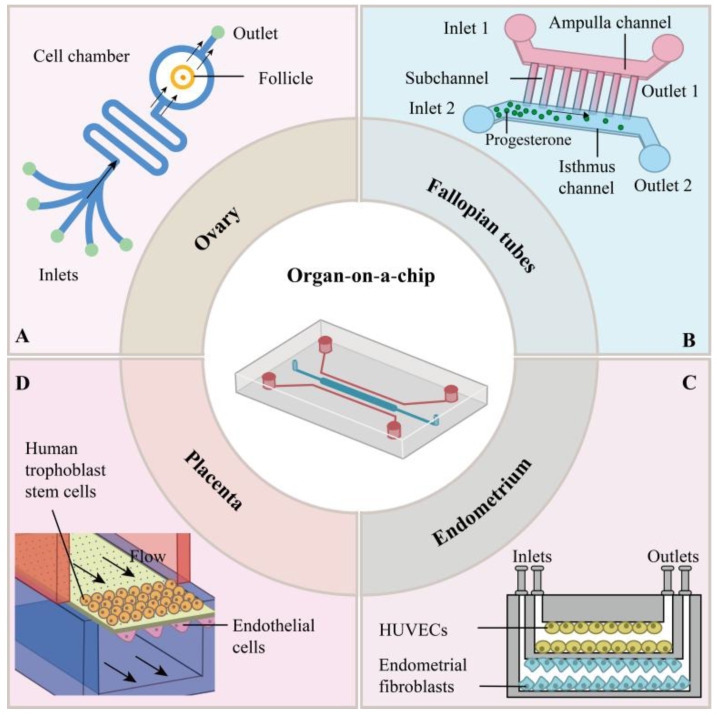
Organ-on-a-chip modeling of female reproductive system. (**A**) Five inlets and one outlet with one chamber where follicle is cultured, combined with three polydimethylsiloxane layers, and an upper polymethyl methacrylate layer [24]. (**B**) 3D schematic of oviduct chip. Main channels are ampulla channel and isthmus channel, respectively. Progesterone is injected into the ampulla channel (Inlet 2) and a small volume of liquid aspirated from the ampulla channel’s outlet (Outlet 2). This operation drives passive diffusion of progesterone along parallel subchannels toward the isthmus channel, thereby forming a concentration gradient [25]. (**C**) Perivascular endometrial stromal microarrays composed of human primary umbilical vein endothelial cells and endometrial stromal cells [26]. (**D**) A bioengineered placental barrier model was constructed in a perfused OOC system. Human trophectodermal stem cells were inoculated on the upper channel, where they could differentiate into cytotrophoblasts and syncytiotrophoblasts and self-assemble into a double-layered trophoblast epithelium with a placental microvillus-like structure under dynamic culture conditions. HUVEC (human umbilical vein endothelial cells) were cultured on the other side of the collagen-coated membrane to mimic the fetal endothelium [27].

To date, research on organ-on-a-chip models of the female reproductive system has primarily focused on key anatomical components, including the ovary, fallopian tube, endometrium, and placenta. Researchers have leveraged OOC technology to simulate the physiological and pathological processes of these organs, with the aim of establishing a biomimetic platform that closely recapitulates the in vivo microenvironment for advancing studies on the female reproductive system. Below are schematic illustrations depicting the simulation of each organ-on-a-chip in the female reproductive system (Figure 1).

### 2.1. Ovarian-Organ-on-a-Chip

The ovaries perform dual roles in reproduction and endocrinology. Structurally, they consist of an outer cortex (housing developing follicles) and an inner medulla (providing vascular support), which are essential for maintaining follicle growth and hormone synthesis. Reproductively, follicular development and ovulation generate ova, with one mature ovum released monthly, approximately 14 days before menstruation [28]. Endocrinologically, they secrete estrogen and progesterone, pivotal for female sexual development, menstrual cycle regulation, and pregnancy sustenance [29].

Ovarian-organ-on-a-chip technology, grounded in microfluidics, mimics the ovarian microenvironment to reproduce physiological and pathological states in vitro. Constructed using methods such as microfluidic encapsulation of diverse ovarian cell types (including oocytes, granulosa cells) and decellularized extracellular matrix scaffolds, these chips enable the simulation of follicular maturation under hormonal gradients and dynamic steroidogenesis resembling the menstrual cycle [30,31,32]. They have been applied to study ovarian diseases, such as the OTME-Chip and ovarian cancer organ-on-a-chip (OvCa-Chip), which mimic the ovarian tumor microenvironment to elucidate tumor metastasis mechanisms and facilitate antimetastatic drug screening [33,34].

#### 2.1.1. Fabrication Methods and Materials

Ovaries consist of oocytes, granulosa cells, mesenchymal stromal cells, and immune cells, etc. Ovarian-organ-on-a-chip is a miniaturized biological model based on microfluidic technology which can reproduce the physiological and pathological state of the ovary in vitro by simulating its microenvironment and function. Ovarian organoids are mainly designed to simulate both reproductive and endocrine aspects of the ovary, and are usually constructed using microfluidic technology, including a microfluidic platform and encapsulated ovarian tissue. 3D-printed microporous scaffolds with precisely engineered pore geometries (e.g., 30°/60° angles) significantly enhance ovarian follicle survival and restore endocrine function, achieving breakthrough progress in anatomical and functional ovarian reconstruction [35]. The isolated and cultured cells are encapsulated into the microfluidic chip, and the different cell types are co-cultured by connecting the various parts through precise assembly technology [30,31]. Choi et al. [36] encapsulated early secondary preluminal follicles in microcapsules consisting of a softer biodegradable collagen (0.5%) hydrogel core and a stiffer slowly degrading alginate (2%) hydrogel shell to produce ovarian microtissues. Follicular development is largely dependent on hormones and nutrients, and disorders of hormones and nutrients can lead to abnormal follicular development, revealing the critical role of mechanical heterogeneity in regulating follicular development and ovulation in mammals.

Most current microfluidic devices are made of optically transparent polydimethylsiloxane (PDMS) [37]; due to the fragility of most cells, the gelatinized microcapsules containing cells should be gentle, and due to the hydrophilic and biocompatible nature of the hydrogel, the encapsulated cells can maintain a high level of viability and normal function for a longer period of time [32]. However, the application in ovarian models is severely limited by the absorption of steroid hormones [38]. Thus, hydrogel-based systems (e.g., gelatin, alginate, dECM) have become the standard, offering superior biocompatibility, minimal hormone absorption for accurate quantification [39], and adequate diffusion properties despite lower gas permeability than PDMS. For ovarian modeling, the need for a biomimetic, low-absorption environment clearly outweighs the fabrication advantages of PDMS. Moreover, in tissue engineering, decellularized extracellular matrix (DECM) has been shown to have great potential to promote regeneration of various organs such as kidney, liver and heart. Ovarian tissue DECM for ovarian tissue engineering has also yielded promising results in isolated follicle transplantation [30]. Pors et al. [40] studied human pre-sinus follicles inoculated from ovarian tissue into DECM. The results showed a high survival rate of follicles injected with Matrigel after 3 weeks of xenografting in mice.

#### 2.1.2. Simulation of the Physiological Environment of the Ovary

The development of follicles is a complex process. In mammals, the follicle is the functional unit of the ovary and provides the microenvironment for oocyte growth and maturation. Ferraz et al. [41] utilized an innovative dynamic microfluidic system to culture in vitro domestic cat and dog follicles enclosed in or isolated from the ovarian cortex. The survival of follicles under different culture conditions was compared, as well as the expression of markers of follicular development and oocyte health. The results showed species-specific and tissue-type-specific differences for microfluidic cultures. Ovarian cortical tissue from domestic cats remained viable under flow, similar to the conventional agarose gel control, but ovarian cortical tissue from dogs did not show similar viability. Preovulatory follicles isolated from both species grew most favorably in conventional alginate bead culture, but the culture system had no effect on follicular development or expression of markers of oocyte health overall. Aziz et al. [24] established a microfluidic chip for culturing individually encapsulated human ovarian follicles. Ovarian follicles were encapsulated in 3D calcium alginate hydrogel beads and then cultured in the chip and Petri dishes under the same conditions. Ovarian follicles cultured on the chip showed successful growth in terms of hormonal trends and diameter increase similar to those cultured in Petri dishes, designing organoids that can be used to culture individual human ovarian follicles (Figure 1A).

On the endocrine side, Xiao et al. [42] constructed a microfluidic system to support mouse ovarian follicles to produce the hormone profile of the human 28-day menstrual cycle, which controls the dynamics of the human female reproductive tract and peripheral tissues in single, dual and multi-unit microfluidic platforms (Solo-MFP, Duet-MFP and Quintet-MPF, respectively). The introduction of dynamic medium flow promoted follicle production of ovarian steroid hormones compared to static follicle culture. Ovarian tissue cultures were then tested in Solo-MFP and Duet-MFP, which showed that these systems support follicular maturation and differentiation and have a similar ovarian hormone secretion profile to the isolated follicle culture method. In summary, ovarian-organ-on-a-chip are constructed using methods such as microfluidic encapsulation and decellularized extracellular matrix scaffolds. These techniques enable the simulation of physiological environments, including follicular maturation under hormonal gradients and dynamic steroidogenesis mimicking the menstrual cycle.

#### 2.1.3. Applications in the Study of Ovarian Diseases

Ovarian-organ-on-a-chip have demonstrated significant utility in modeling ovarian cancer and testing the toxicity of chemotherapeutic drugs (Table 1). Ovarian cancer, particularly its predominant form epithelial ovarian cancer which accounts for over 90% of cases, poses a severe threat to women’s health. A significant clinical challenge is its high mortality rate, largely attributable to the deep pelvic location of the ovaries and the absence of specific early symptoms. This often leads to delayed diagnosis until advanced stages. These complexities underscore the critical need for advanced experimental models that can accurately mimic the ovarian tumor microenvironment to improve our understanding of the disease.

OTME-Chip [33] is a microarray that mimics the ovarian tumor microenvironment, usually consisting of multiple parts, by combining gene editing, next-generation RNA sequencing (RNA-seq) and other technologies to mimic the tumor microenvironment of ovarian cancer patients. The chip consists of platelet-perfused vascular endothelial tissue and adjacent collagenous matrix, enabling precise visualization of the invasive dynamics of the platelet-mediated entry of cancer cells into the tumor microenvironment via endothelial cells. By using OTME-Chip, researchers can gain insights into the mechanism of platelets’ role in the tumor microenvironment and the effect of antitumor–antiplatelet combination therapy, which provides new ideas and methods for the treatment of ovarian cancer [43].

Saha et al. [34] developed a microfluidic chip system called OvCa-Chip to reproduce for the first time in vitro the process of vascular endothelium-mediated platelet extravasation in the microenvironment of ovarian cancer. The research team constructed a 3D co-culture system: the upper layer was a tumor sphere formed by human ovarian clear cell carcinoma cells embedded in stromal gel, and the lower layer was a functional vascular network formed by HUVEC, which was separated by a porous membrane and exerted physiological-grade fluidic shear force. It was found that CXCL12 secreted by cancer cells increased vascular permeability by 2.3-fold and induced a 4-fold increase in the efficiency of platelet transendothelial migration by activating endothelial CXCR4 receptors. This model breakthrough reveals the mechanism of platelet–endothelial–cancer cell tripartite interactions in ovarian cancer metastasis, providing a highly bionic platform for antimetastatic drug screening.

**Table 1:** Organ-on-a-chip models in female reproductive system.

Organ/Chip Type	Construction Methods	Pros and Cons of Materials	Simulated Physiological Environment	Key Applications	References
Ovarian-organ-on-a-chip	-Microfluidic encapsulation (e.g., collagen-alginate hydrogel) -Decellularized ECM (DECM) scaffolds -Co-culture of oocytes, granulosa, and stromal cells	-PDMS Pros: gas permeability Cons: poor absorption of steroid hormones -hydrogel-based systems (e.g., gelatin, alginate, dECM) Pros: adequate diffusion properties Cons: low gas permeability	-Follicle maturation under hormone gradients -Dynamic steroidogenesis mimicking menstrual cycles	-Ovarian cancer modeling (e.g., OTME-Chip, OvCa-Chip) -Drug toxicity testing for chemotherapy agents	[24,30,31,32,36,38,39,40,41,42]
Fallopian-organ-on-a-chip	-Lithography/3D-printed serpentine channels -Primary epithelial cell culture with cyclic fluid shear stress	-PDMS Cons: absorbs hormones and small molecules -Sol–gel coatings: Pros: reduce absorption Cons: compromise permeability and mechanical stability; swelling and cracking	-Ciliary beating under flow conditions -Embryo transport via peristaltic motion	-Infertility mechanism studies -Enhanced embryo development and zygote genome reprogramming	[25,44,45,46,47,48,49,50,51,52,53]
Endometrial-organ-on-a-chip	-Tri-layered “epithelium-stroma-vasculature” microfluidic design -Integration of TEER/pH sensors for real-time monitoring	-PDMS Pros: hormone absorption -hydrogels Pros: gas-permeability advantages with hydrogel biomimicry -Thermoplastics Pros: excellent optics and minimal molecule absorption	-Menstrual cycle phases (proliferative/secretory) -Embryo implantation microenvironment	-Endometriosis pathogenesis (e.g., β-catenin-driven invasion) -Drug screening for endometrial receptivity	[26,51,54,55,56,57,58,59,60,61,62,63,64]
Placental-organ-on-a-chip	-Dual-channel system mimicking maternal–fetal circulation -Co-culture of HUVEC (vascular) and BeWo (trophoblast) cells	-PDMS Cons: absorption of drugs and hormones -Glass Pros: optical clarity and zero absorption Cons: lacks gas permeability -SLA/DLP using PEGDA-based resins Pros: low absorption and gas permeability Cons: low transparency and architectural flexibility	-Nutrient/toxin transport across placental barrier -Hypoxia-induced preeclampsia pathology	-Bacterial infection dynamics (e.g., *E. coli*-triggered inflammation) -Drug transfer studies (e.g., glyburide)	[27,65,66,67,68,69,70,71,72,73,74,75,76]
Multi-organ-chip	-EVATAR platform (integration of ovary, uterus, cervix, liver) -Modular microfluidic interconnections	-	-Hormonal crosstalk (e.g., 28-day menstrual cycle) -Systemic drug metabolism and toxicity analysis	-PCOS studies -Chemotherapy toxicity assessment (e.g., paclitaxel)	[42,77,78]

### 2.2. Fallopian-Organ-on-a-Chip

Crucial to female reproduction, fallopian tubes transport ova from ovaries to the uterus and enable sperm-ovum fusion. Structurally, they comprise fimbriae (for ovum capture), ampulla (fertilization site), isthmus (regulated transport), and intramural segment; functionally, they require ciliary beating, nutrient secretion, and a stable microenvironment for gamete viability. Their structural and functional integrity is essential for fertility; disorders can cause infertility and increase ectopic pregnancy risk, making them a key area of reproductive health research [79,80].

Fallopian-organ-on-a-chip technology uses photolithography or 3D printing to create chips with hollow channels mimicking the tubal lumen, controlling fluid dynamics to maintain cell function and culturing primary epithelial cells to simulate physiological functions like ciliary oscillation [44,45,46]. These chips are applied to study physiological functions, such as embryo transport influenced by mechanical and hormonal factors [47], and address diseases, including creating better in vitro fertilization environments for infertility treatment and exploring ovarian cancer, given its potential origin in fallopian tubes [48,49,50,81].

#### 2.2.1. Fabrication Methods and Materials

The core of constructing a fallopian-organ-on-a-chip is to simulate its anatomical structure, cellular composition, and dynamic microenvironment, with the functions of fallopian tube cilia movement, mucus secretion, and embryo transport. In terms of the structural design of the chip, hollow channels are constructed through photolithography or 3D printing to simulate the morphology of the tubal lumen. It is usually designed as a serpentine or branching structure to match the physiological curvature, and to simulate the flow characteristics of the tubal fluid by precisely controlling the hydrodynamic parameters and material transport, so as to maintain the polarity of the cells and metabolic activity [44,45]. 3D printing was initially employed indirectly to produce complex master molds for PDMS chip fabrication, marking the technology’s introductory phase in this field. Currently, it is transitioning toward direct 3D bioprinting for constructing fallopian tube models [47,61]. Fallopian-organ-on-a-chip models have traditionally used PDMS fabricated via soft lithography [47,61], leveraging its gas permeability and optical clarity for epithelial culture and dynamic imaging [75]. However, PDMS absorbs hormones and small molecules [51], complicating hormonal or drug studies. Common workarounds like medium pre-conditioning [61] or parylene coatings—which reduce absorption but compromise permeability and mechanical stability—are imperfect. Sol–gel coatings also risk swelling and cracking [52,53]. These limitations motivate a shift to absorption-resistant alternatives.

Periodic flow promotes ciliary oscillations, whereas static culture leads to ciliary degeneration. Xiao et al. [42] designed serpentine microchannels to mimic tubal fluid flow through periodic flow rate variations so that the cilia oscillate at a frequency close to physiological levels. In terms of cell source and selection, primary cells can be obtained by isolating epithelial cells from human or animal fallopian tubes, which retains the natural function, but there are difficulties in expansion and batch differences, Sung et al. [46] used primary fallopian tube epithelial cells to successfully maintain cilium activity in microfluidic chip for over 2 weeks. Yu et al. [25] used a microfluidic chip to design a bionic tubal surface, mimicking the topology of tubal epithelial cilia through a micropatterned substrate, combined with a dynamic mucus layer gradient, and used high-speed micro-imaging to track sperm motions in real time and quantitatively analyze the correlation between their evasive behaviors and the surface’s physicochemical characteristics (Figure 1B).

#### 2.2.2. Simulation of the Physiological Environment of Fallopian Tubes

Due to the specificity of the location and morphology of the oviduct, there are fewer studies on in vitro models of the oviduct. The transport function of the oviduct is one of the current research hotspots, and the directional oscillation of the cilia of the oviduct epithelial cells is the key mechanism to drive the transport of ova. Xiao et al. [42] constructed the first human-originated tubal microarray, culturing primary tubal epithelial cells in a microfluidic channel to mimic tubal fluid flow by periodic fluid shear. The cilia in the microchip oscillated at a frequency of 8–10 Hz, compared with only 2–3 Hz in the static culture group. After injection of polystyrene particles with a diameter of 150 μm, the particles travelled up to 70% of the length of the microchip within 48 h, at a rate consistent with the in vivo data. Ferraz et al. [47] developed a bovine oviductal microchip, in which a pneumatic membrane was embedded in the chip to simulate peristalsis of the oviduct, and found that mechanical deformations could facilitate the efficiency of embryo transport. Hormone slow-release microspheres were embedded in the matrix layer to release estradiol and progesterone, respectively, creating a concentration gradient change, and the proportion of embryos cultured in the chip that developed to the blastocyst stage was increased by 25% compared with conventional culture under the dynamic regulation of hormones (Table 1).

#### 2.2.3. Applications in the Study of Fallopian Tube Diseases

Marcia et al. [48] designed a microchip oviduct, cylindrical in shape, with a length of 2 cm and a diameter of 1 cm, which is more compatible in shape with the anatomical features of the isthmus of the fallopian tube. The microchannel of the chip conforms to the anatomical structure of the fallopian tube in shape, and the inner wall is planted with primary epithelial cells of the mouse fallopian tube to simulate the biochemical environment of the fallopian tube. At the same time, the chip was connected to an automatic fluid exchange device to simulate the tubal fluid flow environment. The chip tubal platform supports a more physiological syncytial genetic reprogramming that is closer to the in vivo environment than traditional IVF. The platform could modulate the apical medium to support physiological sperm-ovum interactions and fertilization, completely eliminating polysperm fertilization and ovum orphan activation. In addition, the improved microchip oviduct supports embryonic development to the blastocyst stage, providing an enhanced in vitro environment for syncytial genome reprogramming.

In ovarian cancer research, high-grade plasma ovarian cancer is thought to originate as a precursor lesion within the fallopian tube [49,50]. Tubal microarrays can be used to simulate the microenvironment of the fallopian tube and to study the mechanism of early onset and development of HGSOC. Jo et al. [81] developed an integrated detection platform Extracellular Vesicle High-Throughput Analysis System (EV-HTAS), which combines microfluidic microarray technology with an artificial intelligence algorithm to achieve high-throughput molecular typing and early diagnosis of plasma extracellular vesicles in ovarian cancer patients. The study conducted blind testing on 428 samples and found that the sensitivity of EVs with double-positive CLDN3/HE4 combined with high miR-200c-3p expression for early ovarian cancer diagnosis reached 94.7%, which is significantly better than the traditional CA-125 assay providing a transformative tool for liquid biopsy of ovarian cancer.

### 2.3. Endometrial-Organ-on-a-Chip

The endometrium, a critical uterine inner layer, is indispensable for female reproduction. Structured with functional, basal, and spongy layers [82,83,84], the functional layer undergoes cyclic shedding/regrowth with hormones; the basal layer sustains regeneration. It also needs vascularization for nutrients and regulated cytokine secretion to support embryo adhesion, key for OOC modeling. It remodels under estrogen influence during the peri-implantation phase, adjusting immune tolerance to form an optimal site for embryo implantation and placental growth [85]. As the cornerstone of pregnancy, successful embryo implantation in the endometrium initiates fetal development.

In vitro endometrial-organ-on-a-chip have become a research frontier, designed for diverse purposes such as simulating cyclic changes, studying implantation, exploring diseases, and drug testing. These chips mimic the endometrium’s physiological environment by incorporating vascular networks to replicate blood supply and endocrine functions, and recapitulating menstrual cycle-related hormonal responses. In disease research, they overcome limitations of traditional models, facilitating studies on endometriosis through simulating the estrogen microenvironment and showing potential in exploring endometrial cancer tumor–microenvironment interactions for targeted therapy development [58,59,60].

#### 2.3.1. Fabrication Methods and Materials

The exploration of in vitro models of the endometrium has been an important area of research in reproductive medicine and obstetrics and gynecology in recent years. According to existing studies, uterine organ-on-chips may be classified into several types depending on the purpose of the study, such as models that simulate cyclic changes in the endometrium, those used to study embryo implantation, those used to study uterine diseases, or those used for drug testing. In the construction of endometrial-organ-on-a-chip, 3D bioprinting is a key technology for rebuilding its multi-layered, vascularized structure, enabling the study of dynamic processes such as embryo implantation and decidualization. Ahn et al. constructed a vascularized endometrium-on-a-chip with three distinct layers—epithelium, stroma, and vasculature—that faithfully recapitulated hormonal responses and the process of angiogenesis [55]. By printing perfusable vascular networks, researchers can quantitatively analyze drug permeability and create a functional platform to systematically investigate the cellular and molecular interactions that determine the outcome of embryo implantation.

Endometrial-organ-on-a-chip platforms commonly adopt a hybrid material strategy. The structural housing is often PDMS, benefiting from established multi-channel fabrication to separate epithelial and stromal compartments [55], while hydrogels like collagen or Matrigel recreate the 3D stromal matrix inside [61]. This combines PDMS’s structural and gas-permeability advantages with hydrogel biomimicry. Hormone absorption into the PDMS still remains a major confounder for menstrual cycle studies [62]. Thermoplastics like COC/COP and PMMA are thus gaining traction as chip body alternatives, offering excellent optics and minimal molecule absorption [51] for stable, long-term assays. Their drawbacks include lower gas permeability and more complex bonding processes, making them less ideal for rapid prototyping but superior for scalable production where hormone/dose precision is paramount [63,64].

Moreover, Park et al. [54] constructed a microchip: the endometrial organ-on-a-chip is built on a PDMS microfluidic chip with two interconnected upper and lower microchannels. The lower channel, using collagen I/Matrigel extracellular matrix (ECM), is seeded with primary, hormone-pretreated endometrial epithelial cells (EECs, surface) and stromal cells (ESCs, within ECM) to mimic the “epithelium-stroma” bilayer. The upper channel holds trophoblast cells, with an 8 μm porous membrane enabling cell/signal interaction. The microenvironment is regulated via low-speed medium circulation, 5% hypoxia, and embryonic signaling molecules, while fluorescence imaging and molecular detection are integrated to observe and quantify cell dynamics and functions [54].

Gnecco et al. [26] constructed a compartmentalized human endometrial model by microfluidic chip to simulate the interaction between perivascular stroma and endothelial cells. Methodologically, a dual-channel chip was used to segregate cultured perivascular stromal cells (3D collagenous stroma) from endothelial cells, simulating blood flow by fluid shear, while introducing cyclic mechanical strain to simulate physiological endometrial deformation. A chemokine gradient was established within the chip to induce endothelial cells to migrate in a directed manner and form a vascular-like network, which was combined with real-time imaging to analyze cellular interactions, angiogenesis, and barrier function to achieve a highly bionic reconstruction of the endometrial microenvironment (Figure 1C).

#### 2.3.2. Simulation of the Physiological Environment of Endometrium

The endometrium is a highly dynamic and complex tissue whose physiological environment and functional characteristics change significantly during the menstrual cycle, pregnancy and non-pregnant states. One of the anatomical features of the endometrium is that it has abundant blood flow to ensure adequate oxygen supply, and therefore endometrial OOC often contain vascular networks to mimic the blood supply and endocrine functions of the endometrium. Ahn et al. [55] constructed a device consisting of five microchannels arranged in parallel and separated by an array of microposts, and established a microengineered vascularized endometrial microarray consisting of epithelium, stroma and blood vessels, which recapitulated in vivo endometrial angiogenesis and hormonal responses, showing key features of the proliferative and secretory phases of the menstrual cycle. This provides a new in vitro method for drug screening and drug discovery by mimicking the complex behavior of the human endometrium. Endometrial organoids mimic the physiological environment of the endometrium through microfluidics, including the co-culture of epithelial, mesenchymal and endothelial cells, as well as key processes such as angiogenesis and hormone signaling. The microengineered vascularized endometrial microarray (MVEOC) model established by Ahn et al. [55] is capable of reconstructing the endometrial environment and responding to pro-angiogenic factors and hormonal stimuli, thus providing an important experimental platform for the study of the molecular mechanisms of diseases such as endometriosis. In terms of studying endocrine interactions between the endometrium and other organs, Eisa et al. [56] combined endometrial and ovarian cell microarrays to study the effects of estrogen and progesterone on the endometrium and the feedback of the endometrium on ovarian function. Chang et al. [57] created a uterus-on-a-chip; this system recapitulates core uterine functionalities by simulating the physiological microenvironment, which includes a stable temperature, physiological pH, and gradient-based nutrient delivery. It incorporates dynamic fluid perfusion that mimics the circulation of maternal blood and secretions, replicating mechanical stretching of the uterine wall, and incorporating real-time monitoring of embryo metabolism and morphology. By addressing the limitations of traditional static culture, this platform significantly improves embryo survival rates and developmental synchronization (Table 1).

#### 2.3.3. Applications in the Study of Endometrium Diseases

Endometrial diseases have complex pathological mechanisms, and traditional studies have relied on animal models and 2D cell cultures with limitations such as large species differences and low microenvironmental reduction [8,9]. OOC technology has facilitated the accuracy in simulating disease characteristics by constructing patient-specific tissues in three dimensions. Endometriosis is an estrogen-dependent disease, and the estrogen microenvironment can be simulated on organ-on-chips to study the changes in estrogen receptor expression and the mechanism of the related E3 ubiquitin ligase in the abnormal estrogen activity. Using a human endometrial organoid chip model, a consistent downregulation of ERα alongside upregulation of ERβ, BAG2, and MDM2 in both human endometriosis specimens and mouse models was observed in the study. Furthermore, it also validated that CHIP and MDM2 mediate the degradation of ERβ and ERα, respectively, via the ubiquitin–proteasome pathway, and that targeted interference with BAG2 and MDM2 may elicit therapeutic effects in endometriosis [58].

Gnecco et al. [59] designed a synthetic extracellular matrix (ECM) chip containing endometrial epithelial, stromal, and myometrial cells to mimic the flow of peritoneal fluid through fluid shear. It was found that ectopic endometrial cells showed a 2.5-fold increase in β-catenin nuclear translocation in response to IL-1β stimulation, driving their invasion into the myometrium. The model also revealed that angiogenesis in ectopic lesions was dependent on the VEGF-A/VEGFR2 signaling axis, and that anti-angiogenic drugs reduced vessel density by 60%. In endometrial cancer research, Oksana et al. [60] showed that endometrial organ-on-a-chip excels at simulating endometrial diseases. It recapitulates epithelial-stromal cell layer morphology and spatial organization, and mimics in vivo cyclical physiological processes via perfusion systems. It can simulate menstrual cycle-related molecular regulatory networks, aiding in dissecting mechanisms of endometrial disorders like endometriosis. Also, it optimizes embryo implantation timing to support in vitro fertilization research for infertility linked to endometrial issues, and serves as a platform for personalized drug screening in endometrial cancer, enabling targeted therapeutic exploration.

### 2.4. Placental-Organ-on-a-Chip

The placenta, composed of trophoblast, chorionic and fetal vasculature, features syncytiotrophoblast-formed barrier, cytotrophoblast-mediated implantation, and chorionic villi for exchange [86]. Structurally, its chorionic villi need branching architecture to expand exchange surface; functionally, it requires regulated nutrient/gas transport, hormone secretion (hCG and progesterone), and maternal–fetal immune tolerance—all of which constitute key criteria for the development of placental-organ-on-a-chip. Ethical limitations on in utero placental research necessitate further development of in vitro models.

Placental-organ-on-a-chip, modeled on maternal–fetal interface physiology, use cells like HUVEC and BeWo with microchannel and porous membrane structures to mimic material exchange, hormone secretion, and immune functions [86]. These chips replicate the placenta’s multicellular environment, simulating molecular transport and pregnancy changes [65,66]. In disease research, they help study pre-eclampsia, reveal pathological mechanisms, and screen drugs, while also enabling real-time monitoring of the placental barrier’s infection response [69,70]. Amniotic tissue models further explore inflammation and infection, providing platforms for maternal–fetal health research [72,73].

#### 2.4.1. Fabrication Methods and Materials

Placenta-organ-on-chips are designed to mimic the core functions of the maternal–fetal interface, including material exchange, hormone secretion, and immunity, and their core design is based on the physiological characteristics of the maternal–fetal interface. Femtosecond laser printing has created villi-like hydrogel membranes within microchips to mimic the maternal–fetal interface [87], while DLP bioprinting has enabled 3D patterning of placental cells to model hormone secretion and nutrient transport [88]. Furthermore, high-throughput bioprinting of trophoblast-laden microdroplets has standardized placental organoid production, overcoming the limitation of manual methods and offering a robust platform for drug screening [89].

Blundell et al. [65] simulated maternal meconium blood flow and fetal chorionic blood flow separately through independent microchannels, replicating the physiological gradient of the placental chorion and enabling the simulation of a dual circulatory system. A porous membrane is used to separate the maternal and fetal chambers, allowing small molecules such as glucose and amino acids to diffuse across the membrane to simulate dynamic material exchange, and applying periodic shear stress to simulate hemodynamic stimuli. Placental organoids are usually constructed using HUVEC, which represent the endothelium of the placental vasculature, and BeWo cells, which mimic the function of trophoblast cells, to model the placental barrier. The placental barrier was represented by a collagen-coated membrane with a high concentration of collagen, and the transport of glucose between the two was investigated. In placental chips, BeWo cells are seeded on the upper layer of collagen membranes to mimic the trophoblast layer at the maternal interface, and are induced to fuse into syncytiotrophoblasts via forskolin. HUVECs are seeded on the lower layer to simulate fetal vascular endothelium. Through dynamic perfusion co-culture, a “trophoblast–collagen–vascular” sandwich-like structure is formed, which successfully recapitulates the transplacental glucose transport function [90].

Placental-organ-on-a-chip models face a comparable material challenge. Conventional designs often sandwich a porous thermoplastic membrane between PDMS chambers to separate maternal and fetal sides [39], utilizing PDMS for gas permeability and imaging clarity. However, PDMS absorption of drugs and hormones critically distorts transport kinetics [76], driving a shift to PDMS-free systems. Glass offers optical clarity and zero absorption but lacks gas permeability and is difficult to fabricate [75]. A highly promising alternative is high-resolution 3D printing (e.g., SLA/DLP) using PEGDA-based resins, which provide low absorption, transparency, and architectural flexibility [75]. Although gas permeability is lower and cytocompatibility requires careful leaching, these materials excel for quantitative transport studies by minimizing compound loss.

Meanwhile, in order to mimic the microstructure of the maternal–fetal interface of the placenta, the researchers constructed a microfluidic chip with a multilayer design to simulate the microstructure of the maternal–fetal interface of the human placenta and a flow design to mimic the dynamic environment of the mother, which was used to study the inflammatory response of the placenta to bacterial infections. Cao et al. [27] constructed a self-assembled human placenta model using trophoblast stem cells (TSCs) in a dynamic organ chip. Methodologically, TSCs were inoculated within the 3D matrix of the microfluidic chip, and the dynamic microenvironment of the maternal–fetal interface was simulated by precisely modulating the fluid shear force with periodic mechanical stretching. Combined with gradient growth factor induction, TSCs were induced to differentiate autonomously into syncytiotrophoblast cells and form placental villus-like structures. A multi-channel system was integrated into the chip to monitor nutrient transport, hormone secretion, and barrier function in real time to verify its physiological bionicity and applicability for drug testing (Figure 1D).

#### 2.4.2. Simulation of the Physiological Environment of Placenta

Placenta-organ-on-chips can mimic the multicellular composition of the placenta, including trophoblast cells, endothelial cells, and so on. These cells interact with each other in the chip and together build a microenvironment similar to that of the human placenta. Blundell et al. [66] constructed a placental microarray that recapitulates placental barrier properties, including the ability to transport small and large molecules, as well as placental chorionic trophoblast and mesenchymal cell interactions, to simulate and study the transfer of drugs from the maternal to fetal circulation using a microengineered model of the human placental barrier. Using the gestational diabetes drug glibenclamide as a model compound, it was shown that this microphysiological system could re-establish the exocytotic transporter protein-mediated active transport function of the human placental barrier in order to limit fetal exposure to maternally administered drugs. The placental microarray is also able to simulate the dynamic changes of the placenta during pregnancy, such as the growth and remodeling of placental villi, and changes in the permeability of the placental barrier. Lermant et al. [67] simulated the dynamic environment of the placental barrier by controlling the hydrodynamic conditions. Trophoblast and epithelial cells were planted in the upper and lower layers of the microchannels, respectively, and intercellular adhesion was enhanced by ECM coating. The cells were cultured under dynamic flow conditions to form a continuous monolayer cell barrier (Table 1).

#### 2.4.3. Applications in the Study of Placental Diseases

The health of the placenta, a vital organ connecting the fetus to the mother, is of vital importance to both mother and baby. Pre-eclampsia is a unique multi-system dysfunction in pregnancy, characterized by hypertension, proteinuria, and placental insufficiency, with a complex pathogenesis and a lack of effective treatments, posing a major threat to the health of mothers and babies [68,69]. Rabussier et al. [70] constructed a disease model that was exposed to hypoxic conditions and modulated perfusion flow to induce a pathological environment. This resulted in a reduction in barrier function, hormone secretion, and microvilli, as well as an increase in the number of nuclei, which is characteristic of the pre-eclamptic placenta, contributing to the mechanistic understanding of pre-eclampsia and other placental pathologies associated with hypoxia-ischemia and supporting the development of effective future therapies through targeting and compound screening activities. The chip designed by Zhu et al. [71] was designed with a two-layer microfluidic channel design, simulating maternal circulation (trophoblast layer) and fetal circulation (endothelial cell layer) respectively, with an extracellular matrix layer in the middle to form a 3D structure, and the study used *Escherichia coli* as a model pathogen, which was infected with graded concentrations through the maternal side of the microchannel. Real-time monitoring showed that bacterial infection triggered a significant inflammatory response 6 h later, with a 3–5-fold elevation of TLR4 receptor activation in trophoblast cells, and peak activation of the NF-κB signaling pathway appeared at 12 h post-infection. Cytokine microarray analysis revealed that the secretion of pro-inflammatory factors, such as IL-6, IL-8, and TNF-α, showed a dose-dependent increase up to 20 times of the basal level, which for the first time achieved real-time visual monitoring of the dynamic infection process of the placental barrier.

The amniotic membrane covers the surface of the fetal side of the placenta and consists of amniotic epithelial cells and amniotic mesenchymal cells. The chorionic villi, also known as the chorionic villi, are the main structure of the placenta and are composed of two different cell types: chorionic trophoblast cells and chorionic mesenchymal cells [72]. Richardson et al. [73] developed a novel amniotic OOC model to simulate the complex structure and dynamic physiological environment of amniotic tissues through microfluidic technology, focusing on the interaction mechanisms between amniotic epithelial cells and mesenchymal cells during inflammation and infection. This innovative platform provides a platform for studying preterm birth-related amniotic inflammation, evaluating anti-inflammatory treatment strategies and exploring the mechanisms of cellular dialogue at the maternal–fetal interface. This innovative platform provides a highly bionic in vitro research model. In addition, Yin’s team [74] developed a 3D microengineered amniotic membrane tissue model based on human-induced pluripotent stem cells (hiPSC) for accurately simulating the inflammatory response of the amniotic membrane triggered by bacterial infection. The team constructed a bilayer hydrogel structure by micropatterning: the upper layer differentiated hiPSCs as amniotic epithelial cells, and the lower layer was induced as amniotic mesenchymal cells with a natural extracellular matrix layer (containing laminin/collagen IV) at the interface. The model simulates amniotic fluid circulation through a perfusion system and integrates a mechanical stretching module to reproduce the mechanical stimuli of uterine contraction. The model reveals for the first time the protective mechanism of amniocytes inhibiting the activation of NLRP3 inflammatory vesicles through exosomal miR-223 delivery and verifies that dexamethasone intervention reduces inflammatory factor levels by 75%. This platform provides a highly physiologically relevant in vitro model for studying preterm birth-associated amniotic cavity infections and evaluating anti-inflammatory treatment strategies.

## 3. Joint Multi-Organ-on-a-Chip Study of the Female Reproductive System

The functional regulation of the female reproductive system involves the synergy of multiple organs, and its complexity makes it difficult for traditional in vitro models to fully simulate physiological or pathological processes. In recent years, MOC technology has provided a revolutionary platform for the study of the female reproductive system. By integrating microfluidics, bioengineering and organoid technologies, researchers have successfully constructed physiologically relevant in vitro models of the female reproductive system, which opens up new pathways for research on the mechanisms of gynecological diseases, drug toxicity assessment, and reproductive health management.

### 3.1. Multi-Organ-Chip

**Figure 2:**
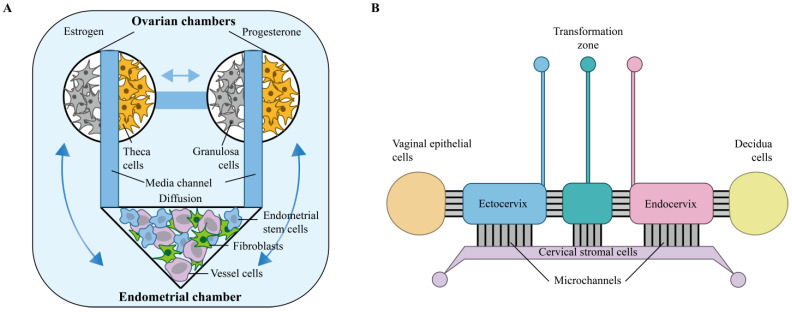
Multi-organ-chip. (**A**) The female reproductive system is composed of interconnected organs, including the uterus and ovaries, which are bidirectionally and precisely regulated by multiple steroid hormones and cytokines. Dual chambers were interconnected by media channels to allow bidirectional endocrine crosstalk between endometrial and ovarian chambers within a chip platform [77]. (**B**) Schematic image of VCD-OOC with different cell culture chambers represented by different colors and connected with each other by an array of microchannels [91]. Blue and pink boxes show ectocervix and endocervix section respectively, connected to vaginal epithelial cells (orange), and vaginal epithelial cells (yellow) respectively, and green box represents squamous columnar transformation zone.

Park et al. [77] successfully developed the world’s first uterus–ovary dual-organ-on-a-chip system by innovatively integrating microfluidics and tissue engineering, which for the first time realized the simulation of bi-directional endocrine communication between the endometrium and ovarian tissues in vitro. This system is the first to achieve in vitro reconstruction of dynamic hormone dialogue between multiple organs of the reproductive system as well as integration of dual regulation of mechanical forces and biochemical signals, which provides a highly bionic platform for mechanistic studies and personalized therapeutic screening of polycystic ovary syndrome, endometriosis, and other diseases (Figure 2A).

In addition, researchers have developed MOC models for the female reproductive system. For example, a six-chamber vagina–cervix–ecdysplasia organ-on-a-chip (VCD-OOC) has been constructed, which is capable of reproducing the female reproductive tract during pregnancy and, for the first time, recreates the microenvironment of the human vagina in vitro in both healthy and dysbiotic states. The microarray contains culture chambers for vaginal epithelial cells, cervical epithelial and stromal cells, and metaphase cells. The microarray revealed that Ureaplasma urealyticum could reach the cervical epithelium and meconium within 48 h without causing cell death, and that infection alone caused less inflammation, whereas co-infection with Lipopolysaccharide (LPS) caused a lot of inflammation. Meanwhile, animal models showed that vaginal inoculation with low doses of Ureaplasma microsporum did not cause preterm labor, and high doses had only a partial effect, whereas intra-amniotic injections caused 67% of preterm deliveries [78,91] (Figure 2B).

Although current co-culture OOC bring a lot of value, there are still some problems that have not yet been solved, with existing models lacking functional vascular networks and relying on passive diffusion for nutrient transport, resulting in co-culture systems that last days (Table 1).

### 3.2. EVATAR

EVATAR [42,78] is the combination of “Eve” and “avatar”, which is an innovative multi-organ co-culture in vitro model that integrates microfluidics, organoids, and bioengineering to enable the study of the female reproductive system with other. The core concept of EVATAR is to build a miniature reproductive system on a chip, which provides a revolutionary platform for the study of hormone regulation, reproductive toxicity, disease mechanisms, and personalized medicine (Table 1).

Xiao et al. [42] used Solo-MFP and Duet-MFP systems with pneumatic drive technology and Quintet-MFP system based on embedded electromagnetic drive technology designed for single and multiple tissue culture respectively. Mouse ovarian tissues were cultured in the Solo-MFP and Duet-MFP systems for 28 days, resulting in follicles producing a 28-day hormonal profile of the menstrual cycle. To test downstream ovarian hormone control in human female reproductive tract and peripheral tissues, ovaries, fallopian tubes, uterus, cervix and liver were cultured in the Quintet-MFP System. To generate hormone-coupled isolated female reproductive tracts, the investigators cultured mouse ovary, human fallopian tube, endometrium, ectocervix, and liver tissues in the Quintet-MFP for 28 days; this combination of tissues within the system came to be known as EVATAR. The system maintains consistent pituitary hormone cycling and encapsulates pituitary hormone control of relevant tissue functions, effectively mimicking the human 28-day menstrual cycle. EVATAR is also important for drug discovery and toxicological studies. It can test the impairment of ovarian function and synergistic toxicity of liver metabolism by chemotherapeutic drugs such as paclitaxel and cisplatin, and has a wide range of applications. By simulating different physiological and pathological states, EVATAR can help scientists better understand the mechanism of drug action in the human body, thus reducing the failure rate in clinical trials.

EVATAR is an innovative technology with a broad future perspective and development potential; this powerful tool allows the integration of hormonal signals between organs in a way that phenotypically replicates the human menstrual cycle and pregnancy.

## 4. Integrated Readouts for Functional Assessment of Female Reproductive OOC Models

To validate the physiological relevance of female reproductive OOC models, a suite of quantitative readouts is employed, increasingly shifting from endpoint analyses to integrated, real-time monitoring [92].

Electrical readout primarily trans-epithelial/trans-endothelial electrical resistance (TEER), are the gold standard for assessing the integrity of barrier-forming tissues. This is particularly critical in placenta- and endometrium-on-a-chip models, where TEER measurements quantify the dynamic tightness of the maternal–fetal barrier or the receptive endometrial lining, respectively [55]. In oviduct-on-a-chip models, TEER values of approximately 400 Ω·cm^2^ have been used to confirm the formation of a polarized, functional epithelial monolayer [93]. However, TEER measurements in microfluidic devices can suffer from high variability and are highly sensitive to even minute gaps in cell coverage, necessitating careful geometric consideration and the integration of fixed electrodes to improve reproducibility and enable continuous monitoring [94].

Chemical readouts provide crucial data on metabolic activity and endocrine function. Hormone assays are paramount; for instance, ovarian-organ-on-a-chip models are validated by measuring secreted estradiol and progesterone via ELISA, with estradiol levels in the range of 50–80 pg/mL indicating functional steroidogenesis in murine models [95]. Similarly, placenta-on-a-chip models demonstrate functionality through the secretion of human chorionic gonadotropin (hCG) [88]. Integrated electrochemical or optical sensors are increasingly used to monitor the cellular microenvironment in real time, tracking parameters like pH and dissolved oxygen (O_2_) [92,96]. The oxygen consumption rate (OCR) serves as a robust indicator of cell viability and metabolic health across all reproductive tissue models [96], while glucose transport assays are a primary method for assessing the barrier function in placental models [39].

Optical readouts offer complementary methods for functional assessment. Barrier permeability is quantified using fluorescent dyes of varying molecular weights, such as Lucifer yellow and FITC-dextran. In placenta- and endometrium-on-a-chip models, these assays yield apparent permeability coefficients (Papp), typically in the range of 10^−5^ to 10^−6^ cm/s, to characterize transport dynamics and barrier integrity [55,97]. For mechanically active tissues, calcium (Ca^2+^) imaging using genetically encoded reporters like GCaMP is a powerful tool. This has been demonstrated in ex vivo uterine models, where Ca^2+^ waves corresponding to myometrial contractions were mapped with frequencies ranging from 0.01 Hz in proestrus to 0.17 Hz in diestrus, providing a functional benchmark for future endometrium-on-a-chip models designed to study contractility [98].

Mechanical readouts are emerging to quantify the forces exerted by or on the tissues. While direct, integrated force sensing is still nascent, mechanical strain is often measured optically by tracking the displacement of fluorescent microbeads embedded within flexible membranes, a technique demonstrated in lung-on-a-chip models that is directly applicable to studying uterine contractions or placental shear stress [99].

## 5. Applications of OOCs for Female Reproductive System Organs

### 5.1. Drug Discovery and Screening

Traditional drug toxicity assessment systems rely on static 2D cell cultures and animal models, which have inherent shortcomings such as insufficient physiological relevance and significant species differences, making it difficult to truly reflect the complex endocrine regulation and inter-organ interactions in the human body. The microfluidic technology-based organoids of the female reproductive system can accurately reproduce key microenvironmental parameters such as tissue–fluid interface, cyclic hormone fluctuations, and extracellular matrix stiffness by constructing a 3D dynamic culture system. Taking endometrial toxicity assessment as an example, the platform can monitor the dynamic changes of stromal cell metaplasia, epithelial barrier function and immunomodulatory factors under drug exposure in real time, and combine with single-cell transcriptome analysis to reveal the mechanism of selective damage to specific cell subpopulations by drugs [100,101].

### 5.2. Modeling of Female Diseases

OOC also provide a new platform for disease research and treatment: For example, bacterial vaginosis is a major cause of preterm births, miscarriages and increased HIV incidence [102,103]. The Wyss Institute for Biologically Inspired Engineering at Harvard University has developed a vaginal microchip designed to study imbalances in the vaginal microbiota, known as bacterial vaginosis. The chip cultures live cells in grooves on a glass chip to mimic the biological activity of the human vagina. The vaginal microchip has already been used to study the effects of bacterial vaginosis on sperm migration, and scientists are working to combine the vaginal microchip with a cervical microchip to study the mechanisms of transmission of viral infections, such as HPV, and a variety of bacterial diseases, which could lead to the development of medicines that can regulate the vaginal microbiota and treat related diseases. In addition, the researchers also plan to simulate the effects of the menstrual cycle and hormonal changes on women’s reproductive health by adding vascular cells and female hormones to the chip, providing new ideas for hormone-related drug research [104].

### 5.3. Promotion of Research in Assisted Reproductive Medicine

The development of assisted reproductive technologies (ART) has been limited by a lack of knowledge of the dynamic interaction mechanisms of the reproductive system, and ART is an important means of addressing infertility, such as IVF and ICSI, for couples who are unable to have children because of obstacles to natural conception [105,106]. By constructing a highly bionic in vitro microenvironment, the female reproductive system organ microarray provides a new research paradigm for revealing key biological processes such as follicle development, embryo transport, and the regulation of the implantation window, which significantly promotes the breakthrough of basic research in assisted reproductive medicine.

Gatimel’s team [107] reproduced for the first time in vitro the specific supportive effect of the fallopian tube microenvironment on sperm motility by constructing a model of the human fallopian tube organ. The team used primary fallopian tube epithelial stem cells cultured in Matrigel in three dimensions to form an organoid with ciliated and secretory functions, whose tip forms a microcystic lumen and continuously secretes key components of the tubal fluid. This model breaks new ground in simulating the microenvironment of sperm capacitation, providing a highly bionic platform for studying the physiology of fertilization, developing novel in vitro fertilization culture systems and assessing reproductive toxicity.

Eisa et al. [56] elaborated on the synergistic and innovative application of stem cell technology and organ-on-chip technology in the treatment of human infertility. The researchers pointed out that the combination of hiPSC-differentiated germ cells and microfluidic organ-on-chips could accurately simulate key reproductive events such as follicular development, spermatogenesis and embryo implantation. By constructing an ovarian chip, the maturation rate of oocytes in vitro was successfully increased from 35% in traditional culture to 68%. Breakthrough cases showed that endometrial chips implanted with patients’ autologous endometrial stem cells can reproduce the genetic fluctuations associated with the menstrual cycle and accurately predict individual differences in response to ovulation-promoting drugs. This technology system provides a transformative tool for personalized fertility treatment, in vitro production of gametes and reproductive toxicity assessment.

## 6. Challenges and Future Prospects

### 6.1. Challenges and Limitations

Female reproductive system organ-on-chips are highly dependent on progenitor cells (e.g., endometrial cells, follicular cells) to mimic physiological functions [108]. However, primary cell acquisition requires invasive procedures (e.g., endometrial biopsy or ovarian tissue removal) with limited cell viability and functional maintenance. Although induced pluripotent stem cells (iPSC) and organoid technologies offer alternatives for cell sources, their differentiation efficiency and maturity are still insufficient. For example, ovarian organoids have not yet been able to fully mimic the hormonal regulatory network of follicular development, resulting in lower oocyte maturation rates in vitro than in vivo [109]. In addition, stem cell-derived cells may carry epigenetic abnormalities that affect the accuracy of drug toxicity assessments [6].

The female reproductive system involves the synergistic action of multiple organs such as ovaries, fallopian tubes and uterus, and its microarrays require the integration of heterogeneous cells and 3D tissue structures in a microfluidic system [110]. Endometrial microarrays need to contain epithelial cells, stromal cells, and vascular networks, while ovarian microarrays need to mimic the 3D microenvironment of follicular development [111]. In the prior art, long-term cell survival within the chip (>28 days) is still limited by uneven nutrient gradients and accumulation of metabolic wastes [112]. In addition, mechanical force simulation is prone to fatigue of the chip structure or cell detachment. The study shows that the dynamic flow control system combined with elastic hydrogel material can partially improve the structural stability, but the precise regulation of mechanical parameters still needs a breakthrough [42].

In terms of materials, microfluidic chips are mostly fabricated using soft lithography (e.g., PDMS molding), and the most notable drawback of PDMS is its strong adsorption of hydrophobic substances (e.g., proteins, hydrophobic drugs) [53]; this may result in lower than expected actual drug concentrations in the microarray or distort the quantitative test results of cellular secretions. Therefore, how to overcome the adsorption of hydrophobic substances by PDMS is an important research direction at present [113,114]. In addition, the chip designs of different laboratories vary significantly, and the lack of uniform validation standards hinders data comparability. In addition, the miniaturization of microarrays is in contradiction with the demand for high throughput, and the current models have low throughput, making it difficult to meet the demand for drug screening [115].

### 6.2. Directions for Improvement and Innovation

Organ-on-chips may become a major trend in future research. Based on the above deficiencies, the technology of OOC can break through at the following levels to carry out technical paths to improve chip stability. Most OOC studied to date rely on the cultivation of a single organ. However, the female reproductive system constitutes an intricate regulatory network, rendering single-organ chip simulations inadequate in accurately replicating physiological processes [116]. Existing models (EVATAR, VCD-OOC, et al.) have made progress by integrating multiple organs including the ovary, uterus, and liver. Nevertheless, future development should focus on expanding to more complex systems, such as the placenta–fetus interface and immune system interactions. For instance, the FMi-PLA-OOC model investigates the mechanisms of inflammatory signaling and drug transport across the placental barrier through the connection of maternal and fetal tissues. Such advanced platforms will facilitate the elucidation of molecular mechanisms underlying pregnancy complications, including pre-eclampsia [117].

Due to the shortcomings of PDMS, the development of new materials with low adsorption properties is crucial. These materials can reduce the non-specific adsorption of hormones and drugs, thereby improving the accuracy and reliability of experimental results [118,119]. Additionally, the development of degradable materials holds significant promise as it enables dynamic tissue remodeling during long-term culture, which is essential for maintaining the physiological relevance of the chip models over extended periods [120].

Traditional OOC exhibit limitations in long-term dynamic monitoring. Since the physiological environment of the human body is characterized by continuous dynamic regulation, traditional models fail to provide continuous and dynamic data, which significantly hinders real-time monitoring capabilities in studies of specific physiological conditions. To address this, the adoption of modular chip design is recommended, as it can reduce costs while enhancing experimental repeatability [121]. Furthermore, combining 3D printing technology with biomaterials and incorporating bioprinting techniques allows for the construction of endometrial models with layered structures, which can effectively simulate epithelial–mesenchymal interactions, thereby improving the physiological relevance of the chip models [122].

In conclusion, although OOC technology for the female reproductive system has made remarkable progress, it still faces several challenges and limitations. The dependence on specific progenitor cells restricts the long-term stability of the models, as primary cells are invasive and difficult to expand, while induced pluripotent stem cell-derived cells lack maturity. Existing microarrays are insufficient in simulating complex microenvironments, lacking functional vascular networks and having short-lived co-culture systems. Issues such as the non-standardization of materials and fabrication processes also affect data reproducibility and comparability. However, the future of this technology is promising. To overcome these obstacles, future research should focus on developing biomimetic materials to reduce non-specific adsorption, optimizing multi-organ coupling systems to better mimic physiological processes, and innovating high-throughput detection technologies. By addressing these aspects, OOC technology is poised to revolutionize female reproductive health research, enabling the development of more physiologically relevant models and paving the way for precision medicine approaches in improving women’s health.
